# Efficacy of two vitamin D immunoassays to detect 25-OH vitamin D2 and D3

**DOI:** 10.1016/j.plabm.2019.e00130

**Published:** 2019-07-29

**Authors:** Emily Garnett, Jieli Li, Deepthi Rajapakshe, Estella Tam, Qing H. Meng, Sridevi Devaraj

**Affiliations:** a Department of Pathology & Immunology, Baylor College of Medicine and Texas Children's Hospital, Houston, TX, USA; b Department of Pathology & Immunology, UT MD Anderson Cancer Center, Houston, TX, USA

**Keywords:** 25-Hydroxyvitamin D, Vitamin D2, Immunoassay, Recovery, Assay comparison

## Abstract

**Objectives:**

Assessment of Vitamin D status by measurement of 25-Hydroxyvitamin D (25-OH-D) is widely performed by immunoassay. Yet, the ability of these assays to detect Vitamin D2 (as 25-OH-D2) or Vitamin D3 (as 25-OH-D3) varies. It is important to recognize the ability of an assay to quantitate either form of 25-OH-D to evaluate Vitamin D status of supplemented patients. We evaluated detection of 25-OH-D2 and 25-OH-D3 by two assays in our medical center.

**Design and methods:**

The Abbott Architect i1000 SR 25-OH Vitamin D assay and Roche Cobas 8000 Vitamin D assay were compared for their recovery of 25-OH-D2 or D3 from spiked serum samples. Samples with known endogenous concentrations of 25-OH-D2 or D3 by LC-MS/MS were also measured to calculate bias between our assays and LC-MS/MS.

**Results:**

Recovery of 25-OH-D3 in spiked samples was similar by Architect (84–87%) and Cobas (90%). Recovery of 25-OH-D2 was lower than 25-OH-D3, and was poorer by Architect (37–40%) than by Cobas (69–71%). In measurement of samples with known 25-OH-D concentrations, performance of Architect and Cobas assays was similar for 25-OH-D3. However, at concentrations >50 nmol/L 25-OH-D2, the Architect assay exhibited large average negative bias (−27%).

**Conclusions:**

While the Architect and Cobas assays performed similarly in detection of 25-OH-D3, the Architect assay was significantly poorer at detecting 25-OH-D2 than Cobas, with poorer recovery and significant negative bias at higher concentrations of 25-OH-D2. This agrees with other studies, and indicates that caution should be used in interpreting Architect 25-OH-D results in patients supplemented with Vitamin D2.

## Highlights

• We compared Architect and Cobas total 25-OH-D assays for detection of 25-OH-D2 or D3.
• The Architect assay exhibited poor recovery of 25-OH-D2 and negative bias relative to LC-MS/MS.
• Architect and Cobas assays performed similarly for detection of 25-OH-D3.

## 1 Introduction

Measurement of 25-Hydroxyvitamin D (25-OH-D) is commonly performed to assess Vitamin D status in clinical laboratories. Vitamin D can be synthesized in skin from 7-dehydrocholesterol following ultraviolet light exposure, which produces Vitamin D3 (cholecalciferol). Vitamin D2 (ergocalciferol) is present in plant sources, and can be absorbed from the diet. Both D2 and D3 are subsequently converted to 25-OH-D by 25-hydroxylase activity in the liver. Renal conversion of 25-OH-D to 1,25-OH-D by 1-alpha hydroxylase is required to produce the active form of Vitamin D, but 25-OH-D concentration reflects the total Vitamin D reservoir and is thus the recommended analyte for Vitamin D status assessment.

Vitamin D supplementation is common. The two forms of 25-OH-D [25-OH-D2 (D2) and 25-OH-D3 (D3)] have essentially the same physiological activity despite their slight structural differences. While most supplements are provided as Vitamin D3, patients taking Vitamin D2 supplements may be encountered, and thus, measurement of both 25-OH-D2 and 25-OH-D3 is crucial to accurately assess Vitamin D status.

The most common method of assay for 25-OH-D is FDA-approved immunoassay [1], but recovery of 25-OH-D2 is generally lower than that of 25-OH-D3, and the degree to which these assays recognize 25-OH-D2 is highly variable [2,3]. Therefore, it is important to verify how individual assays perform to enable accurate determination of Vitamin D status in this supplemented population.

We evaluated the performance of our institution's total 25-OH-D assay on the Abbott Architect i1000 SR platform to detect either 25-OH-D2 or 25-OH-D3. Samples spiked with either 25-OH-D2 or 25-OH-D3 and reference laboratory samples with either 25-OH-D2 or 25-OH-D3 concentrations determined by LC-MS/MS were used for evaluation. As a comparison to the Architect assay, samples were also tested on the Roche Cobas 8000.

## 2 Materials and methods

### 2.1 Analytical methods

Samples were tested using the Architect 25-OH Vitamin D assay (chemiluminescent microparticle immunoassay) on the Abbott Architect i1000 SR instrument, and the Cobas Vitamin D assay (electrochemiluminescence binding assay) on the Roche Cobas 8000 instrument.

### 2.2 Sample preparation and study design

Spiked samples were prepared from serum samples previously tested at our institution for total 25-OH-D. These samples were pooled for analysis into low [< 50 nmol/L (<20 ng/mL), n = 8], moderate [75–122 nmol/L (30–49 ng/mL), n = 7], or high [> 125 nmol/L (>50 ng/mL), n = 7] 25-OH-D. Prior to analysis, samples were spiked with 130 nmol (50 ng) 25-OH-D2 or 25-OH-D3, or methanol as a solvent control to 1% of the final sample volume. 25-OH-D2 and 25-OH-D3 stock solutions were purchased from Millipore-Sigma (cat# 740223 and 739669) and working solutions were made in methanol. Three replicates were performed for spiked samples. Samples with known concentrations of 25-OH-D2 or 25-OH-D3, determined by quantitative LC-MS/MS, were purchased from ARUP Laboratories, and were tested in singlicate.

### 2.3 Statistics

Recovery of 25-OH-D was calculated for spiked samples by:

$$recovery=\frac{\left[spiked\right]\left(nmol/L\right)-\left[unspiked\right]\left(nmol/L\right)\frac{volume\;plasma}{total\;volume}}{25-OH-D\;added\;\left(nmol/L\right)}\times 100.$$

Percent bias was calculated by

$$\%\;bias=\frac{test\;method\;result-LC-MS/MS\;result}{LC-MS/MS\;result}\times 100.$$

Paired t-tests were performed in Microsoft Excel, and graphs were rendered in GraphPad Prism. Results from recovery studies performed in triplicate are expressed as mean ± standard deviation.

## 3 Results

### 3.1 Recovery studies

Recovery studies for 25-OH-D2 or 25-OH-D3 were performed by measurement of 25-OH Vitamin D in samples spiked with 25-OH-D2 or 25-OH-D3. Recovery of 25-OH-D3 in spiked samples was 87 ± 10% for the low pooled sample, 84 ± 2% for the moderate pooled sample, and 86 ± 4% for the high pooled sample on the Architect assay, and was 69 ± 10% for the low pool and 71 ± 15% for the moderate pool on the Cobas assay. Recovery of 25-OH-D2 was poorer, at 40 ± 5%, 39 ± 5% and 37 ± 4% on the Architect assay, and 69 ± 10% for the low pool and 71 ± 15% for the moderate pool on the Cobas assay (Table 1). Recovery for the high pooled samples was unable to be calculated for Cobas because measurements exceeded the linear range of the assay.

**Table 1:** Recovery studies of 25-OH-D with spiked samples by Architect or Cobas assays.

Sample	25-OH-D species	[25-OH-D] added, nmol	Architect			Cobas		
			Measured [25-OH-D], nmol/L	Expected [25-OH-D], nmol/L	Recovery	Measured [25-OH-D], nmol/L	Expected [25-OH-D], nmol/L	Recovery
Low	none	0	35.38 ± 2.1			40.86 ± 3.3		
Low	D2	130.93	86.81 ± 7.0	166.30 ± 2.1	40 ± 5%	130.47 ± 11.7	171.79 ± 3.8	69 ± 10%
Low	D3	134.35	151.75 ± 14.5	169.72 ± 2.1	87 ± 10%	161.42 ± 14.7	175.21 ± 3.8	90 ± 12%
Moderate	none	0	82.56 ± 7.3			97.72 ± 3.6		
Moderate	D2	130.93	132.94 ± 11.6	213.49 ± 7.3	39 ± 5%	189.56 ± 17.1	228.64 ± 3.1	71 ± 15%
Moderate	D3	134.35	194.63 ± 9.9	216.91 ± 7.3	84 ± 2%	217.12 ± 19.8	232.06 ± 3.1	90 ± 14%
High	none	0	151.68 ± 4.2			183.68 ± 15.1		
High	D2	130.93	198.19 ± 2.3	282.62 ± 4.2	37 ± 4%	>250	314.60 ± 18.4	a
High	D3	134.35	265.50 ± 6.1	286.03 ± 4.2	86 ± 4%	>250	318.02 ± 18.4	a

a Unable to calculate recovery due to measured values above instrument reportable range.

### 3.2 Bias comparison to LC-MS/MS

**Fig. 1:**
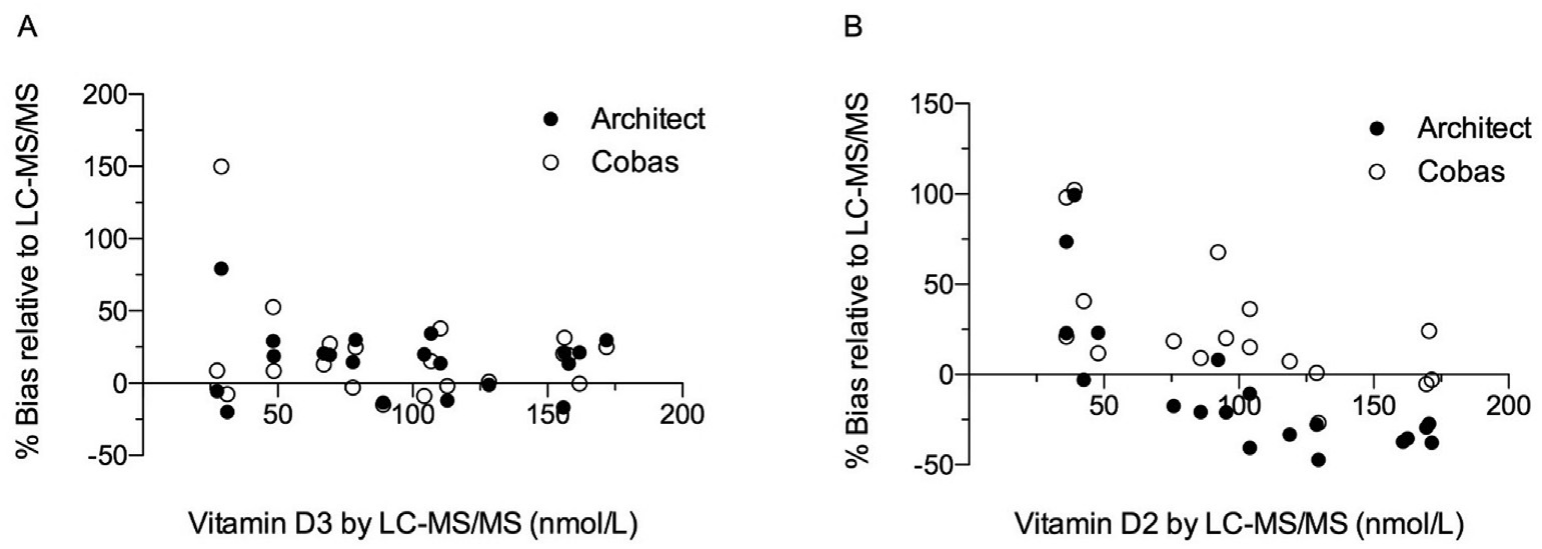
**Bias plots of total 25-OH-D assay results relative to 25-OH-D2 or D3 by LC-MS/MS**. Samples were measured in singlicate. Percent bias was calculated for Architect and Cobas assays for measurements of samples with A) known 25-OH-D3 or B) known 25-OH-D2 concentrations.

Samples with 25-OH-D2 or 25-OH-D3 previously quantitated by LC-MS/MS were used to evaluate these findings in the context of known concentrations of endogenous 25-OH-D. For samples with known concentrations of 25-OH-D3, the Architect and Cobas assays both measured higher total 25-OH-D than LC-MS/MS (Fig. 1A). Proportional bias observed for samples with <50 nmol/L (<20 ng/mL) 25-OH-D3 did not differ between the two assays: Relative to LC-MS/MS, median bias was 40.6% by Architect, and 8.7% by Cobas (n = 5, p = 0.17). For samples with >50 nmol/L (>20 ng/mL) 25-OH-D3, median bias was 12.1% for Architect and 15.1% for Cobas (n = 12, p = 0.90).

For samples with concentrations of 25-OH-D2 < 50 nmol/L (<20 ng/mL), both Architect and Cobas assays measured higher total 25-OH-D than LC-MS/MS, and bias did not differ between assays (Fig. 1B). The Architect assay exhibited median proportional bias of 23.0% relative to LC-MS/MS, while Cobas exhibited median bias of 18.6% (n = 5, p = 0.30). Yet, the Architect assay measured lower concentrations of total 25-OH-D relative to LC-MS/MS for higher known 25-OH-D2 concentrations (Fig. 1B). In samples with known 25-OH-D2 > 50 nmol/L (>20 ng/mL), median proportional bias was −40.9% for Architect, whereas it was 19.5% for Cobas (n = 15, p = 3 × 10^−7^).

## 4 Discussion

This study examines the ability of the Architect and Cobas total 25-OH-D assays to detect either 25-OH-D2 or 25-OH-D3. We report poor recovery of 25-OH-D2 by the Architect assay in spiked samples, and corroborate this finding with low detection of total 25-OH-D in samples with known high concentrations of 25-OH-D2.

We observed that the Cobas assay has superior ability to detect 25-OH-D2 as compared to the Architect assay (median recovery of 65% vs. 37%), while the ability of the two assays to detect 25-OH-D3 is similar (median recovery of 74% vs. 81%). The two assays operate on different principles for 25-OH-D detection, which could explain this disparity. The Architect assay uses an anti-Vitamin D polyclonal goat IgG as a detection method, while the Cobas assay relies on a ruthenium-labeled Vitamin D binding protein [4,5].

While previous work has suggested that spiked 25-OH-D behaves dissimilarly from endogenous 25-OH-D in assays [6], we observed similar behavior of the two tested assays in the detection of 25-OH-D2 from spiked samples and in samples with endogenous 25-OH-D quantitated by LC-MS/MS. Total 25-OH-D concentrations were not provided for ARUP samples, and only 25-OH-D2 or 25-OH-D3 concentration was reported for each sample, so recovery as a percentage of total 25-OH-D cannot be calculated. Yet, the disparity in measured 25-OH-D by Architect for samples with known high 25-OH-D2 and 25-OH-D3 concentrations mirrors the trends seen in spiked samples. Samples with high endogenous concentrations were reported to contain >150 nmol/L 25-OH-D2 or D3, which is likely to represent a large proportion of the total 25-OH-D stores and thus increases our confidence in this comparison.

The Architect 25-OH Vitamin D assay package insert claims mean cross-reactivity with 25-OH-D2 as 82%, and with 25-OH-D3 as 105% [4]. Using the method in the insert, calculated median cross-reactivity for both 25-OH-D2 (37%) and 25-OH-D3 (85%) was lower than indicated for the Architect assay. This is consistent with reports from other investigators [7,8]. The Cobas Vitamin D assay also performed more poorly than manufacturer specifications. The Cobas assay package insert claims 92% cross-reactivity with 25-OH-D2 and 100% cross-reactivity with 25-OH-D3 [5]. However, calculated median cross-reactivity was 67% for 25-OH-D2 and 87% for 25-OH-D3.

At our institution, adult reference ranges used for classification of Vitamin D status are <50 nmol/L (<20 ng/mL) for deficiency, 53–73 nmol/L (21–29 ng/mL) for insufficiency, and 75–200 nmol/L (30–80 ng/mL) for sufficiency, with a cutoff value of 53 nmol/L (21 ng/mL) to indicate sufficiency in pediatric patients. Our results suggest that the Architect assay could misclassify the Vitamin D status of some patients that are receiving supplementation with Vitamin D2. In LC-MS/MS-quantified samples with 75–150 nmol/L (30–60 ng/mL) D2, four of the ten samples would have been classified as Vitamin D insufficient by Architect measurements, while all ten samples would be correctly classified by the Cobas assay.

The cutoff value used for Vitamin D toxicity at our institution is 375 nmol/L (150 ng/mL). While no samples measured by LC-MS were near this cutoff, a 25-OH-D2-spiked sample tested on the Architect assay contained 282 nmol/L (112.8 ng/mL) 25-OH-D and was measured at 198.3 nmol/L (79.3 ng/mL). This suggests that despite the poor recovery of 25-OH-D2 by the Architect assay, misclassification of a toxic concentration of Vitamin D2 as normal is unlikely. However, values measured above the normal range that do not meet the cutoff for toxicity could result in missed cases of Vitamin D2 overdose.

## 5 Conclusions

This study confirms earlier findings that the Abbott Architect 25-OH Vitamin D assay performs more poorly in detection of 25-OH-D2 than indicated by the manufacturer, and also more poorly than the Roche Cobas assay. While the Cobas assay performed better than Architect, it still did not meet manufacturer specifications. Based on this, we suggest caution in the interpretation of Vitamin D status in patients who are receiving supplementation with Vitamin D2, and highlight the need for manufacturers to improve assay performance for quantification of both forms of Vitamin D.

## Conflicts of interest

Authors report no conflicts of interest.

## References

[1] Wyness S.P., Straseski J.A. (2015). Performance characteristics of six automated 25-hydroxyvitamin D assays: mind your 3s and 2s. Clin. Biochem..

[2] Freeman J., Wilson K., Spears R. (2015). Performance evaluation of four 25-hydroxyvitamin D assays to measure 25-hydroxyvitamin D2. Clin. Biochem..

[3] Garg U. (2018). 25-Hydroxyvitamin D testing: immunoassays versus tandem mass spectrometry. Clin. Lab. Med..

[4] (2013). Abbott ARCHITECT 25-OH Vitamin D Assay [directional Insert 3L52, G3-3094/R05] (Package Insert).

[5] (2015). Roche Cobas 25-Hydroxyvitamin D Assay [directional Insert 2015-05, V 6.0 English] (Package Insert).

[6] Carter G.D., Jones J.C., Berry J.L. (2007). The anomalous behaviour of exogenous 25-hydroxyvitamin D in competitive binding assays. J. Steroid Biochem. Mol. Biol..

[7] Cavalier E., Carlisi A., Bekaert A.C. (2012). Analytical evaluation of the new Abbott Architect 25-OH vitamin D assay. Clin. Biochem..

[8] Brock A.T., Strickland S.W., Bazydlo L.A.L. (2017). An underestimation of 25-OH vitamin D in patients with renal disease by the Abbott Architect immunoassay. J. Appl. Lab. Med..

